# Validation of a new simple scale to measure symptoms in heart failure from traditional Chinese medicine view: a cross-sectional questionnaire study

**DOI:** 10.1186/s12906-016-1306-7

**Published:** 2016-09-02

**Authors:** Tieh-Cheng Fu, Yi-Chung Lin, Ching-Mao Chang, Wei-Ling Chou, Pei-Hsun Yuan, Min-Hui Liu, Chao-Hung Wang, Juei-Chao Chen, Hen-Hong Chang, Tai-Long Pan

**Affiliations:** 1 Department of Physical Medicine and Rehabilitation, Chang Gung Memorial Hospital, No. 222, MaiJin Road, Anle District, Keelung, 20401 Taiwan; 2 Graduate Institute of Traditional Chinese Medicine, College of Medicine, Chang Gung University, No. 259, Wen-Hwa 1st Road, Kwei-Shan, Taoyuan, 33302 Taiwan; 3 Center for Traditional Medicine, Taipei Veterans General Hospital, No.201 Sec. 2, Shipai Road, Beitou District, Taipei City, 11217 Taiwan; 4 Department of Traditional Chinese Medicine, Chang Gung Memorial Hospital, No. 222 MaiJin Road, Anle District, Keelung, 20401 Taiwan; 5 Heart Failure Center, Department of Internal Medicine, Chang Gung Memorial Hospital, Keelung, College of Medicine, Chang Gung University, Tao-Yuan, No. 222 MaiJin Road, Anle District, Keelung, 20401 Taiwan; 6 Department of Statistics and Information Science, Fu Jen Catholic University, No. 510 Zhongzheng Road, Xinzhuang District, New Taipei City, 24205 Taiwan; 7 Research Center for Chinese Medicine & Acupuncture, and School of Chinese Medicine, College of Chinese Medicine, China Medical University, No. 91 Hsueh-Shih Road, North District, Taichung City, 40402 Taiwan, Republic of China; 8 Graduate Institute of Clinical Medical Sciences, College of Medicine, Chang Gung University, No. 259, Wen-Hwa 1st Road, Kwei-Shan, Taoyuan, 33302 Taiwan; 9 School of Traditional Chinese Medicine, Chang Gung University, Taoyuan, Taiwan; 10 Liver Research Center, Chang Gung Memorial Hospital, Taoyuan, Taiwan; 11 Department of Medical Research, China Medical University Hospital, China Medical University, Taichung, Taiwan

**Keywords:** Inquiry, Traditional Chinese medicine, Heart failure, Aerobic capacity

## Abstract

**Background:**

Current clinical practices used to functionally classify heart failure (HF) are time-consuming, expensive, or require complex calculations. This study aimed to design an inquiry list from the perspective of traditional Chinese medicine (TCM) that could be used in routine clinical practice to resolve these problems.

**Methods:**

The severity of documented HF in 115 patients was classified according to their performance in maximal exercise tests into New York Heart Association (NYHA) functional classification (FC) II or NYHA FC III. Concomitantly, the patients were assessed using the new TCM inquiry list and two validated quality of life questionnaires, namely, the Short Form 36 (SF-36) generic scale and the Minnesota Living with Heart Failure Questionnaire (MLHFQ). Factor analysis was applied to extract the core factors from the responses to the items in TCM inquiry list; logistic regression analysis was then used to predict the severity of HF according to the extracted factors.

**Results:**

The TCM inquiry list showed moderate levels of correlation with the physical and emotional components of the SF-36 and the MLHFQ, and predicted the functional class of HF patients reliably using logistic regression analysis, with a correct prediction rate with 64.3 %. Factor analysis of the TCM inquiry list extracted five core factors, namely, Qi Depression, Heart Qi Vacuity and Blood Stasis, Heart Blood Vacuity, Dual Qi-Blood Vacuity, and Yang Vacuity, from the list, which aligned with the perspective of TCM as it relates to the pattern of HF. The correct prediction rate rose to 70.4 % when Dual Qi-Blood Vacuity was combined with the MLHFQ. The excessive false-negative rate is a problem associated with the TCM inquiry list.

**Conclusions:**

The TCM inquiry list is a simple scale and similar to patient-reported subjective measures of quality of life in HF, and may help to classify patients into NYHA FC II or NYHA FC III. Factor 4 addresses dizziness, dizzy vision and general weakness, which are critical parameters that distinguish between NYHA FC II and NYHA FC III. Incorporating these three items into the management of HF may help to classify patients from a functional perspective.

**Electronic supplementary material:**

The online version of this article (doi:10.1186/s12906-016-1306-7) contains supplementary material, which is available to authorized users.

## Background

Heart failure (HF) is common and its impact on a patient’s quality of life (QoL) depends on its severity [1]. Finding simple, cheap, and effective ways to evaluate the severity of HF is important. The severity of HF is usually classified according to the patient’s medical history and perceived effort tolerance, which forms the basis of the New York Heart Association (NYHA) functional classification (FC) of HF. Affective interference in the part of patients and cognitive impressions formed by physicians render this approach fairly subjective, especially with regard to NYHA FC II and NYHA FC III. Although a more precise classification based on cardiopulmonary exercise tests (CPET) was proposed by Fu et al. in 2011 [2], this method may be clinically impractical, because of feasibility and cost considerations. Furthermore, assessing a patient’s QoL using questionnaires, including the Short Form (SF)-36, is time-consuming in routine clinical practice because of the large amounts of time required for their completion and the subsequent complex analyses. A simple and cheap “bedside” tool that provides a composite measure of the symptoms and their effects on a patient’s QoL would be useful to describe the severity of the clinical syndrome in individual patients.

Physicians who practice traditional Chinese medicine (TCM) use four diagnostic approaches, i.e., inspection, auscultation and olfaction, interrogation, and palpation to record patients’ information in relation to specific patterns [3, 4]. TCM offers a simple, cheap, and non-invasive approach for evaluation of the severity of HF and does not involve use of expensive equipment. There are several specific patterns associated with the clinical presentation of HF from the perspective of a comprehensive approach that uses TCM [5–9]. Irrespective of these patterns, the main manifestations of HF include palpitations, dyspnea, and fatigue [10, 11]. “Panting on exertion” is used to grade the severity of HF according to NYHA FC [10, 12]. An integrated approach comprising the SF-36, Minnesota Living with Heart Failure Questionnaire (MLHFQ), and a TCM model, which incorporates a TCM inquiry list, could provide another method for evaluating the severity of HF. Furthermore, this integrated method could identify critical parameters that may differentiate between NYHA FC II and NYHA FC III.

The aim of this study was to design an inquiry list from the perspective of TCM (called the TCM inquiry list) that could be used in routine clinical practice to resolve the current problems associated with the FC of HF.

## Methods

### Study aim

The aim of this study was to design a TCM inquiry list that could be used in routine clinical practice to resolve the current problems associated with the FC of HF.

### Ethical considerations

This investigation was performed in accordance with the Declaration of Helsinki and was approved by the Institutional Review Board of Chang Gung Memorial Hospital, Taiwan (103-1515B). All of the subjects provided written informed consent after receiving an explanation of the experimental procedures.

### Overview

A TCM inquiry list was created based on the signs and symptoms of HF found in textbooks and reports. Eligible candidates, who had been classified as NYHA FC II or NYHA FC III based on aerobic capacity during CPET, were enrolled to participate in pre-testing of the TCM inquiry list. These subjects were also assessed using the TCM inquiry list and two validated QoL questionnaires, namely, the SF-36 generic scale and the MLHFQ. The TCM inquiry list was validated using the SF-36 and MLHFQ, which are two accepted tools that measure patient-perceived symptom severity and QoL impairment, and have been used in previous studies of HF [13, 14]. Finally, the anthropometric and exercise performance data from these patients were statistically analyzed, and an integrated SF-36, MLHFQ, and TCM model in the form of the TCM inquiry list was used to identify the critical parameters that differentiate between NYHA FC II and NYHA FC III.

### Development of the TCM inquiry list

An inquiry list was developed as a disease-specific questionnaire to be used when HF patients visit clinics to see TCM physicians. The TCM inquiry list was designed to be simple and self-explanatory for people without a medical background or medical knowledge. Furthermore, the patients were able to complete the questionnaire themselves with little assistance from medical professionals. Each item on the list allowed the patients to respond in a way that reflected the frequency of their symptoms, as follows: 1 = never, 2 = rarely, 3 = sometimes, 4 = frequently, and 5 = always, and in accordance with the severity of their symptoms, as follows: 1 = absent, 2 = mild, 3 = moderate, 4 = severe, and 5 = catastrophic, to facilitate evaluation of the different levels of severity. Data about all items potentially related to the TCM pattern were collected [11]. The prototype of the TCM inquiry list was reviewed by experienced clinicians, including cardiologists and TCM physicians. To fulfil the first criterion of being a simple and self-explanatory list, some items that required professional experience to complete were excluded from the list. Finally, 20 items were retained from the original inquiry list (Table 1). Each patient’s responses were recorded on the TCM inquiry list and the scoring system was based on a patient’s responses to questions about the severity and frequency of their HF symptoms. Hence, if a patient’s response to question (Q) 1 had a severity score of 2 and a frequency score of 3, the score for Q1 was 2 × 3, i.e., 6. The total score for a patient’s responses to the questions on the TCM inquiry list was the sum of the scores from Q1 to Q20.

**Table 1:** TCM inquiry

	Frequency					Severity				
	Never	Rarely	Sometimes	Frequently	Always	No	Mild	Moderate	Severe	Catastrophic
	1	2	3	4	5	1	2	3	4	5
1. Feeling the earth spinning or shaking like on the boat (Dizziness)										
2. Feeling of being lightheaded, woozy, or unbalanced, and see stars (Dizzy vision)										
3. Feeling short of breath when you lie down (Night panting)										
4. Short of breath walking on the flat road in 5 min (Panting on exertion)										
5. Slight dyspnea of physical activity or when resting (Short of breath)										
6. Feeling not enough breath when speaking and need to take a deep breath. (Shortage of qi)										
7. Feel the heart beat suddenly become faster or slower (Palpitation)										
8. Oppression in the chest (Oppression in the chest)										
9. Pain felt anywhere in the chest area (Chest pain)										
10. Feeling cold in the extremities when cold (Cold limbs)										
11. Feel weakness in the arms and legs (Lack of strength in the limbs)										
12. Feeling sick and hardly think (Listlessness of essence-spirit)										
13. Feeling tired easily want to take a rest during a activity (Exhausted)										
14. No desire for motion (Fatigue)										
15. Feeling cold and wear more clothes than the others (Fear of cold)										
16. Forgetful; having a bad memory (Amnesia)										
17. Emotional instability have no patience for everything (Vexation and agitation)										
18. A relatively permanent state of worry and nervousness (Anxiety and preoccupation)										
19. Feel repress motions (Depression)										
20. Feel weakness and no desire to speak (Disinclination to talk)										

*TCM* Traditional Chinese Medicine

### Subject enrollment and eligibility criteria

Arrindell et al. [15] proposed that the items to be tested should be assessed five times, and that at least 100 subjects are required to establish the reliability of this 20-item questionnaire. Hence, this study enrolled 115 patients who had been diagnosed with HF at the Heart Failure Center, Department of Cardiology, Keelung Chang Gung Memorial Hospital, Taiwan. HF was diagnosed by a cardiologist as NYHA FC II or NYHA FC III. The patient exclusion criteria were as follows: a history of life-threatening ventricular arrhythmias, presence of recent unstable angina, occurrence of a myocardial infarction or coronary revascularization within 4 weeks of the start of the study, and presence of uncontrolled diabetes mellitus, severe chronic obstructive pulmonary disease, symptomatic cerebrovascular disease within the past 12 months, collagen vascular disease, alcohol or drug abuse during the previous 12 months, and significant renal or hepatic disease. The participants received optimal HF treatment for at least 4 weeks in accordance with the American Heart Association/American College of Cardiology guidelines and the center’s case management program.

### Data collection

The baseline evaluation of each patient included completion of the preliminary TCM inquiry list, an MLHFQ, an SF-36 questionnaire, and a maximal exercise test using metabolic gas analysis (MasterScreen® CPX; Cardinal Health, Würzburg, Germany), a non-invasive cardiac output monitor (NICOM®; Cheetah Medical, Inc., Wilmington, DE, USA), and near infrared spectrometry (OxyMon®; Artinis Medical Systems, Elst, The Netherlands) to measure the ventilatory and hemodynamic parameters, including minute ventilation, oxygen consumption (VO_2_), carbon dioxide production, peak exercise oxygen consumption (VO_2_ peak), cardiac output, and changes in tissue oxygenation (Δoxyhemoglobin [ΔO_2_Hb], Δdeoxyhemoglobin [ΔHHb]), and the Δtotal hemoglobin concentration [ΔTHb]) in the left frontal cortex region and the vastus lateralis muscle, using standardized protocols previously described by Fu et al. [2].

### Statistical analysis

First, the patients’ anthropometric and exercise performance data were analyzed. An independent *t*-test was used to compare quantitative variables between groups. The chi-squared test and Fisher’s Exact test were used to compare categorical variables between groups. Pearson’s correlation coefficient was used to determine the presence of correlations between scores on the TCM inquiry list and several parameters associated with functional capacity, including QoL and maximal exercise capacity. The inquiry list was investigated by factor analysis to explore possible patterns from the TCM perspective. The ability of the TCM inquiry list to predict HF from a functional perspective was evaluated using logistic regression analysis. Finally, the critical elements needed to distinguish between NYHA FC II and NYHA FC III in a population with HF were determined and possible correlations between these factors and latent physiologic traits were investigated. All of the statistical analyses were performed using IBM®SPSS® version 22.0 software (IBM Corporation, Armonk, NY, USA).

## Results

This study enrolled 115 HF patients who were divided into NYHA FC II (*n* = 66) and NYHA FC III (*n* = 49) groups according to their performance on the maximal exercise test. Patients in the NYHA FC II group had slight (>5 to ≤7 metabolic equivalent [MET]) limitations to their physical activity, and those in the NYHA FC III group had marked (>1.5 to ≤5 MET) limitations to their physical activity. The characteristics of the groups are shown in Table 2. There were no differences between the groups with respect to sex, height, weight, body mass index, resting heart rate, blood pressure, or left ventricular ejection fraction. The clinician’s NYHA-based classification could not determine a patient’s actual functional status and limitations because of the ambiguity of the criteria used to categorize HF patients as NYHA FC II or NYHA FC III. There was a statistically significant difference between the groups for patient age, and all other parameters, including exercise performance and QoL questionnaire and TCM inquiry list scores, showed significant differences between the groups after adjusting for age.

**Table 2:** Summary of demographic, CPET and QoL Data (*N* = 115)

	NYHA FC II	NYHA FC III
Anthropometrics (Clinical characteristics)		
Subjects (N (%))	66 (57)	49 (43)
Gender (M/F)	56/10	38/11
Median age (y) (interquartile range)	56 (50–62)*	62 (57–71)
Height (cm)	166.4 ± 7.6	163.1 ± 8.5
Weight (kg)	71.0 ± 14.9	71.1 ± 19.1
BMI (kg/m2)	25.5 ± 4.4	26.6 ± 6.3
HR (beats/min)	73 ± 11	71 ± 12
SBP (mmHg)	123 ± 22	122 ± 22
DBP (mmHg)	77 ± 13	72 ± 15
NYHA Fc (from clinician)	2.6 ± 0.5	2.5 ± 0.5
Echocardiography		
LVEF (%)	43.5 ± 14.1	39.5 ± 13.6
Exercise performance at peak phase		
Work-rate (Watts)	115 ± 27*	75 ± 22
HR (beats/min)	144 ± 21*	118 ± 22
MBP (mmHg)	115 ± 17*	102 ± 15
V_E_ (L/min)	61.0 ± 14.7*	46.1 ± 14.0
VO_2peak_ (mL/min/kg)	20.7 ± 3.7*	14.0 ± 2.3
V_E-_ VCO_2_ slope	31.25 ± 4.67*	37.14 ± 8.58
OUES	709.1 ± 173.1*	525.8 ± 164.4
Quality of Life		
SF-36		
PCS	51.44 ± 6.73*	45.26 ± 8.54
MCS	48.52 ± 9.40*	42.64 ± 11.10
MLHFQ	16.5 ± 12.9*	25.3 ± 19.2
TCM inquiry	50.6 ± 29.6*	76.0 ± 53.2

Independent *t*-test; *p < 0.05

*CPET* cardiopulmonary exercise test, *Fc II, Fc III* New York Heart Association functional class II, and III, *QoL* quality of life, *BMI* Body Mass Index, *HR* heart rate, *SBP* systolic blood pressure, *DBP* diastolic blood pressure, *LVEF* left ventricular ejection fracture, *MBP* mean blood pressure, *V*_*E*_ minute ventilation, *VO*_*2*_ oxygen consumption, *V*_*E-*_ *VCO*_*2*_ *slope* the slope of minute ventilation vs. carbon dioxide production, *OUES* oxygen uptake efficiency slope, *SF-36* short form 36, *PCS* physical component score, *MCS* mental component score, *MLHFQ* Minnesota Living With Heart Failure Questionnaire, *TCM* traditional Chinese medicine

### Internal consistency and reliability of the TCM inquiry list

The TCM inquiry list showed a high level of internal consistency (Cronbach’s alpha = 0.913). The test-retest-reliability coefficient indicated excellent reliability (R^2^ = 0.967). Both of these parameters demonstrated the stability of the TCM inquiry list.

### Functional correlations

Table 3 presents the correlations between functional capacity parameters and the TCM inquiry list. The score from the TCM inquiry list showed a moderate positive correlation with the MLHFQ (*r* = 0.622; *P* < 0.001), a moderate negative correlation with the physical component score (PCS) of the SF-36 (*r* = -0.532; *P* < 0.001) and the mental component score (MCS) of the SF-36 (*r* = -0.463; *P* < 0.001), and a mild negative correlation with peak VO_2_ (*r* = -0.202; *P* = 0.04). These findings were consistent with the original design of the TCM inquiry list in which a higher score indicated greater severity. The rates at which each test correctly predicted NYHA FC II and NYHA FC III are shown in Table 3. The peak VO_2_ achieved a correct prediction rate of 100 %, because patients were assigned to the NYHA FC II group or the FC III group based on their aerobic capacity. Correct prediction rates for the PCS of the SF-36, the MCS of the SF-36, the MLHFQ, and the TCM inquiry list were 63.5, 60.0, 60.0, and 64.3 %, respectively. The TCM inquiry list had the best prediction rate of all of the questionnaires used in this study.

**Table 3:** The Pearson correlation coefficient of peak VO2, 2 questionnaire and TCM inquiry

	VO_2peak_	PCS	MCS	MLHFQ	TCM inquiry	Correct rate (%)
						NYHA Fc prediction
VO_2peak_	--	0.411 (0.005)	0.251 (0.025)	−0.272 (0.001)	−0.202 (0.04)	100
PCS		--	0.195 (0.037)	−0.538 (<0.001)	−0.532 (<0.001)	63.5
MCS			--	−0.662 (<0.001)	−0.463 (<0.001)	60.0
MLHFQ				--	0.622 (<0.001)	60.0
TCM inquiry					-	64.3

Pearson’s correlation ( ) is the P value

*VO*_*2*_ oxygen consumption, *TCM* traditional Chinese medicine, *PCS* physical component score of short form 36, *MCS* mental component score of short from 36, *MLHFQ* Minnesota Living with Heart Failure Questionnaire, *NYHA Fc* New York Heart Association functional class

### Exploratory factor analysis

Exploratory factor analysis was conducted to scrutinize the TCM inquiry list and to identify any critical parameters in this list that might distinguish between NYHA FC II and NYHA FC III patients. A value of 0.864 on the Kaiser-Meyer-Olkin test indicated adequate correlation matrices. The Bartlett sphericity test was significant at *χ*^2^ = 1363 (*P* < 0.0001), indicating the presence of significant correlations and reinforcing the relevance of the factor analysis.

Exploratory factor analysis extracted five factors without any limit on the possible number of factors in this analysis, for which five-factor varimax rotation explained 69 % of the total variance (Table 4). All of the items in each of these factors had high factor loadings in excess of 0.5, and the low loadings associated with other factors did not exceed 0.4.

**Table 4:** Exploratory factor analysis of TCM inquiry

Factor	Number	Item	Factor loading	Extraction sums of squared loadings	% of varaince	Cumulative %	Cronbach’s α
Qi Depression (Factor 1)	Q18	Anxiety and preoccupation	0.867	8.297	41.487	41.487	0.929
	Q19	Depression	0.833				
	Q20	Disinclination to talk	0.789				
	Q17	Vexation and agitation	0.740				
	Q3	Night panting	0.518				
Heart Qi Vacuity and Blood Stasis (Factor 2)	Q4	Panting on exertion	0.775	1.825	9.124	50.611	0.793
	Q8	Oppression in the chest	0.725				
	Q9	Chest pain	0.708				
	Q5	Shortness of breath	0.690				
	Q6	Shortage of qi	0.644				
	Q7	Palpitations	0.635				
Heart Blood Vacuity (Factor 3)	Q12	Listlessness of essence-spirit	0.672	1.452	7.258	57.869	0.767
	Q14	Fqtigue	0.632				
	Q16	Amnesia	0.565				
Dual Qi-Blood Vacuity (Factor 4)	Q2	Dizzy vision	0.829	1.238	6.189	64.058	0.639
	Q1	Dizziness	0.744				
	Q11	Lack of strength in the limbs	0.569				
Yang Vacuity (Factor 5)	Q10	Cold limbs	0.823	0.989	4.947	69.005	0.802
	Q15	Fear of cold	0.817				

Q13 had excluded (factor loading <0.4 in all factors)

### Logistic regression analysis

All of the factors on the TCM inquiry list were included in the regression model for prediction of NYHA FC. Factors 2 and 4 were incorporated into the prediction model, and only factor 4 yielded a statistically significant difference (*P* = 0.025; Table 5). The total correct prediction rate for NYHA FC II or NYHA FC III increased to 66.1 %.

**Table 5:** Results of the multivariable analyses (stepwise forward p_in_ < 0.05; p_out_ > 0.1)

Variable	Value	β-coefficient	95 % CI of Exp(B)	*p*-value
TCM inquiry				0.661 (correct rate)
Factor 1	continuous	Excluded from the model	-	0.189
Factor 2	continuous	0.409	0.985 ~ 2.299	0.750
Factor 3	continuous	Excluded from the model	-	0.175
Factor 4	continuous	0.478	1.063 ~ 2.450	0.025
Factor 5	continuous	Excluded from the model	-	0.413
Total				
MLHFQ physical + Factor4		1.764	2.431 ~ 14.000	<0.001

*TCM* traditional Chinese medicine, *MLHFQ* Minnesota Living with Heart Failure Questionnaire

**Fig. 1:**
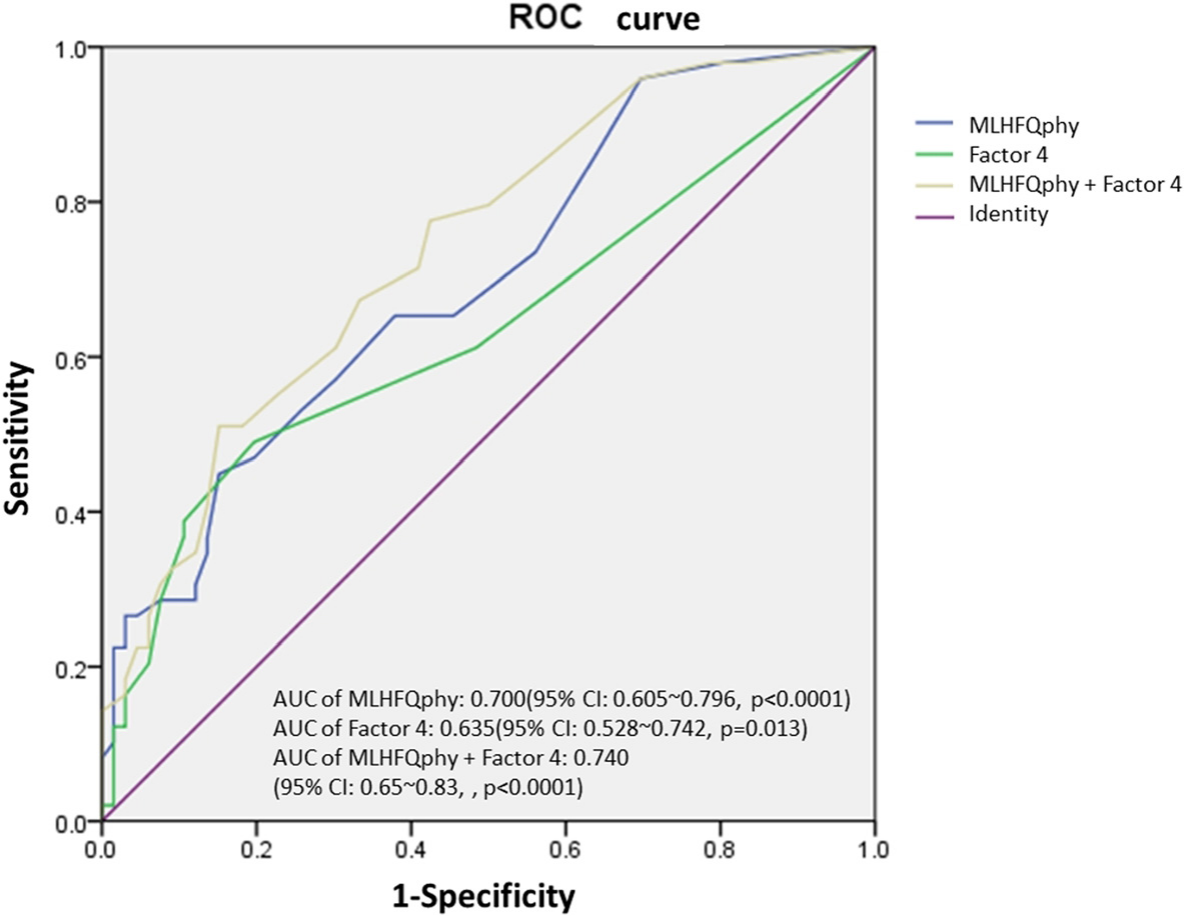
The Receiver Operating Characteristic (ROC) curve of cut-off points in MLHFQ physical component and factor 4 of TCM inquiry to predict NYHA function class. A cut-off point with 9.5 in the score of *MLHFQ physical component,* 1.5 in the score of factor 4 of TCM inquiry and 11.5 in combined both items had the largest diagnostic discriminatory power to distinguish NHYA Fc II and III*.* (*MLHFQphy: physical component of Minnesota Living with Heart Failure Questionnaire*)

To assess and compare the operative characteristics of the MLHFQ and the TCM inquiry list at distinguishing between NYHA FC II and NYHA FC III patients, receiver operating characteristic curves were plotted. The area under the curve (AUC) was largest when the PCS of the MLHFQ and the score for factor 4 on the TCM inquiry list were combined. The AUC was 0.740 (95 % confidence interval: 0.65–0.83; *P* < 0.0001) when 11.5 was used as the cutoff value for this novel parameter, a value that had the greatest diagnostic power to distinguish between NYHA FC II and NYHA FC III (Fig. 1). All candidate variables were excluded from the predictive model. Using this model, the total correct prediction rate for the NYHA FC was 70.4 %, with a sensitivity of 84.8 % and a specificity of 51.0 % (Table 6).

**Table 6:** Association between observed and predicted NYHA function class according to the final model

		Prediction		
		NYHA Fc		Correct %
Observation		II	III	
NYHA Fc	II	56	10	84.8
	III	24	25	51.0
				70.4

Sensitivity: 84.8 %; Specificity:51.0 %; false-positive rate: 30 %; false-negative rate: 28.6 %

*NYHA Fc* New York Heart Association functional class

## Discussion

The ambiguity of the criteria used to determine an HF patient’s classification as NYHA FC II or NYHA FC III and the large economic burden associated with the exercise tests used to distinguish between these classifications prompted the development of a TCM inquiry list that could be used in routine clinical practice. The findings of this study demonstrate that the TCM inquiry list for HF correlated well with the mental and physical aspects of the QoL assessed using the SF-36 and MLHFQ. When combined with the MLHFQ, this novel TCM inquiry list had a correct prediction rate of 70.4 % and could distinguish between NYHA FC II and NYHA FC III.

The five factors extracted from the TCM inquiry list using exploratory factor analysis were Qi Depression (factor 1), Heart Qi Vacuity and Blood Stasis (factor 2), Heart Blood Vacuity (factor 3), Dual Qi-Blood Vacuity (factor 4), and Yang Vacuity (factor 5), as shown in Table 4. These factors are partially compatible with the patterns associated with the clinical perspective of TCM in the HF population, namely, Heart-Lung Qi Vacuity, Dual Vacuity of Qi and Yin, Heart-Kidney Yang Vacuity, and Qi Vacuity with Blood Stasis [11]. However, Yang Vacuity Water Flood, Phlegm-Damp Obstructing the Lung, and Exhaustion of Yin and Desertion of Yang did not emerge in the current analysis, and these factors may result in selection bias [11]. Only factors 1–4 showed significant differences between NYHA FC II and NYHA FC III (Table 7), and can, therefore, be used to distinguish between NYHA FC II and NYHA FC III. Factor 5, which hints at Yang Vacuity, should be present in patients with more severe HF, for example, NYHA FC IV [7]. The HF patients in this study were classified into NYHA FC II and NYHA FC III; hence, factor 5 could not show a significant difference between these groups. The patterns that describe Yang Vacuity Water Flood, Phlegm-Damp Obstructing the Lung, and Exhaustion of Yin and Desertion of Yang did not emerge in the present analysis, and these patterns may also be present in patients with severe HF [5–9]. Without any prediction or interference, the factor analysis essentially extracted these five factors, which are partially compatible with the current TCM pattern (or “Zheng”). This result could in turn confirm the existence of a TCM pattern.

**Table 7:** The difference of the factor score between Fc II and III

	Fc II	Fc III	Effect size	Power
TCM inquiry	50.6 ± 29.6	76.0 ± 53.2*	0.59	0.87
Factoc 1	11.7 ± 9.8	18.7 ± 21.2*	0.42	0.72
Factoc 2	12.3 ± 9.0	18.9 ± 15.1*	0.53	0.82
Factoc 3	7.9 ± 5.5	11.9 ± 9.9*	0.50	0.80
Factoc 4	6.1 ± 5.1	10.2 ± 8.6*	0.58	0.86
Factoc 5	8.7 ± 9.1	11.7 ± 10.3	0.31	0.37

Independent *t*-test * *p*<0.05

*Fc II, Fc III* New York Heart Association functional class II, and III, *TCM* traditional Chinese medicine

When the NYHA FC II and NYHA FC III categories were used as binary variables and the five TCM factors were entered into the logistic regression model to predict NYHA FC, only factor 4 was significantly associated with differences between NYHA FC II and NYHA FC III. Hence, factor 4 is the critical parameter from the perspective of TCM that can help to differentiate between NYHA FC II and NYHA FC III in a population with HF.

The typical clinical manifestations of HF include dyspnea on exertion, nocturnal dyspnea, and leg swelling, and inquiries about these manifestations often comprise parts of the current questionnaires. Factor 4, which includes Q1 (dizziness), Q2 (dizzy vision), and Q11 (general fatigue), and hints at Dual Qi-Blood Vacuity, which is the key pattern associated with coronary artery disease in TCM [7, 8], comprises distinct themes within TCM that are derived from the typical clinical manifestations of HF. Moreover, these three items are the key elements that are associated with the risk of falls [16, 17]. Hence, we considered that integrating this factor from the TCM inquiry list into other questionnaires may increase the prediction rate of HF severity. Since the SF-36 is a structured questionnaire with a specific scoring system, we chose the MLHFQ for integration, and when the PCS from the MLHFQ and the score from factor 4 on the TCM inquiry list were combined, the correct prediction rate rose to 70.4 %, as expected.

Table 8 presents the associations between the score from factor 4 on the TCM inquiry list and the physiologic parameters obtained during the exercise tests, which extracted the latent physiologic traits associated with factor 4. The factor 4 score had a significant negative correlation with peak VO_2_, peak workload, VO_2_ at the anaerobic threshold, oxygenated hemoglobin levels in muscle at the anaerobic threshold, total cerebral hemoglobin level at peak exercise, and the oxygen uptake efficiency slope, and very weak but significant positive correlations with cerebral and muscle deoxygenated hemoglobin levels during peak exercise. The deoxygenated state of the brain and the low cerebral blood flow during the peak exercise phase may be associated with dizziness and dizzy vision. The deoxygenated state of the muscles during the peak exercise phase may be associated with limb weakness. This indicates that the differences between NYHA FC II and NYHA FC III are associated with the blood supply to the brain and muscles, which is in agreement with previous studies [2, 18]. Cardiologists rarely ask patients about dizziness and vertigo when they are assessing the severity of HF, so these items could be integrated into future clinical evaluations of HF severity.

**Table 8:** Pearson’s coefficient of factor 4 score with physiologic parameters

	r (*p*-value)
Peak oxygen consumption	-.266(0.004)**
Peak cardiac output	-.065(0.490)
LVEF	-.067(0.478)
Peak_workload	-.244(0.008)**
VO2 at AT level	-.196(0.047)*
AT_C_O_2_Hb	-.040(0.690)
AT_C_HHb	-.017(0.864)
AT_C_THb	-.040(0.685)
AT_M_O_2_Hb	-.224(0.023)*
AT_M_HHb	.004(0.967)
AT_M_THb	-.092(0.357)
Peak_C_O_2_Hb	-.229(0.014)*
Peak_C_HHb	-.230(0.013)*
Peak_C_THb	-.195(0.037)*
Peak_M_O_2_Hb	-.061(0.518)
Peak_M_HHb	-.184(0.049)*
Peak_M_THb	-.096(0.308)
V_E_-VCO_2_ slope	.131(0.164)
OUES	-.213(0.022)*

Pearson correlation, *p < 0.05 **p < 0.01

*AT* at anaerobic threshold level, *Peak* at peak level, *C* Cerebral, *M* Muscle, *O*_*2*_ *Hb* Oxyhemoglobin, *HHb* deoxyhemoglobin, *THb* total hemoglobin, *V*_*E-*_ *VCO*_*2*_ *slope* the slope of minute ventilation vs carbon dioxide production, *OUES* oxygen uptake efficiency slope

This study has several limitations. Our HF population had similar PCS scores, but lower MLHFQ and worse MCS scores than those reported by other researchers [19, 20]. Weaker associations between peak VO_2_, the MLHFQ score, and the MCS were also noted. Poor correlations between MLHFQ score and CPET parameters have been described in a previous study [21]; however, the correlations between the PCS, MCS, and MLHFQ (Table 3) were similar to those determined in other studies [19, 20, 22]. Possible explanations for this phenomenon are as follows. First, the populations in previous studies were younger and novel NYHA FC criteria were used, namely, the aerobic capacity derived from CPET. Second, the status of patients recruited for the current study was not consistent, and some had undergone exercise training, which may have been a source of bias when the data were analyzed [23, 24].

Q13 was removed from the factor analysis. That means that there is room for improvement in the choice of items included in this inquiry list. The Q13 item was removed from the exploratory factor analysis because of insufficient loading (<0.5 or less) in all factors, or excessive loading (>0.5 or more) in an excessive number of factors (>2 or more). For our purposes, this implies that either Q13 is not an important or common item or that there are surrogate items for Q13 that can be used instead. Therefore, subjects may choose other items first. Deletion of Q13 in this study did not change the final result. Hence, it was reasonable to delete this item.

The correlation coefficients demonstrated relationships between the factors, aerobic capacity, and QoL. We found that the factors in the TCM inquiry list had moderate correlations with the QoL questionnaires, but only weak correlations or no correlations at all with aerobic capacity. This may mean that aerobic capacity did not actually correlate with the TCM inquiry list and the QoL; hence, a non-linear relationship may have existed between QoL and the symptoms associated with the TCM inquiry list. Therefore, further analysis of the data is needed. In addition, the sample size in this study was barely adequate for development of a questionnaire, but should not be considered unsatisfactory [15]. Moreover, we integrated the MLHFQ and the TCM inquiry list scores, because calculating the SF-36 score requires several mathematical procedures, and the total MLHFQ score only required the sum to be calculated. Finally, use of this novel TCM inquiry list to predict NYHA FC was associated with acceptable sensitivity, but the specificity, or false-positivity, was unsatisfactory. In clinical practice, high false positivity leads to a waste of medical resources, while elevated false negativity increases the medical risk. However, sacrificing specificity is reasonable in the high-risk practice of HF management. Further refinements would be necessary before implementing this TCM inquiry list. First, the questions for each item should be designed in such a way that they reflect the status of the patient. For instance, the question we designed for panting on exertion, i.e., “short of breath after walking on the flat road for 5 min” is not adequate to distinguish between NYHA Fc II and NYHA Fc III. “Short of breath on climbing two flights of stairs” may be more appropriate. Second, the patient’s response should be checked by an experienced health care professional to confirm the response is accurate [25]. Third, including three of the other TCM diagnostic tools, i.e., inspection, palpation, and examination by listening and smell, could enable a more comprehensive evaluation.

## Conclusion

The TCM inquiry list is a simple scale and similar to patient-reported subjective measures of quality of life in HF, and may help to classify patients into NYHA FC II or NYHA FC III. Factor 4 addresses dizziness, dizzy vision and general weakness, which are critical parameters that distinguish between NYHA FC II and NYHA FC III. Incorporating these three items into the management of HF may help to classify patients from a functional perspective.

## Additional file

Additional file 1: Data bank. (XLSX 82 kb)

## Abbreviations

BMI: Body mass index
CPET: Cardiopulmonary exercise test
DBP: Diastolic blood pressure
HF: Heart failure
HHb: Deoxyhemoglobin
HR: Heart rate
LVEF: Left ventricular ejection fracture
MBP: Mean blood pressure
MCS: Mental component score
MET: Metabolic equivalences
MLHFQ: Minnesota Living With Heart Failure Questionnaire
NYHA (FC): New York Heart Association (functional class)
O_2_Hb: Oxyhemoglobin
OUES: Oxygen uptake efficiency slope
PCS: Physical component score
QoL: Quality of life
SBP: Systolic blood pressure
SF-36: Short form 36
TCM: Traditional Chinese medicine
THb: Total hemoglobin
V_E-_ VCO_2_ slope: The slope of minute ventilation vs. carbon dioxide production
V_E_: Minute ventilation
VO_2_: Oxygen consumption
